# Insanity from Drugs

**Published:** 1882-05

**Authors:** H. H. Kane

**Affiliations:** DeQuincy Home, Fort Washington, New York City


EDITORS MEDICAL JOURNAL AND EXAMINER: Will you kindly publish these questions in the next issue of your journal, and, if possible, call attention to them editorially? Sincerely yours,

H. H. KANE.

### INSANITY FROM DRUGS.

Members of the medical profession, especially those having had dealings with the insane, are earnestly requested to answer the following questions, fully, yet concisely. The subject is one of so much importance, medico-legally and otherwise, and so very little is to be found upon it in works on insanity, that it merits the attention asked for it.

1. Have you ever seen any cases of insanity, temporary or permanent, or any deviation from the normal mental or moral state that could be traced directly to the use of a single large dose, or the continued use of *opium, or any of its preparations or alkaloids ?*

2. Of what type was such insanity ? Give symptoms.

3. State patient's age, sex, civil condition and occupation.

4. What was its duration and result ?

5. State color of patient's hair, eyes and complexion.

6. Was there any hereditary tendency to insanity, or any history of alcholism, grave nervous disease or any drug habit in the patient's ancestors ?

7. What amount of the drug was used, and for how long a time ?

8. What line of treatment was pursued ?

9. Please answer the same questions regarding the use of *chloral hydrate.*

10. Please answer the same questions regarding the use of *bromide of potassium*, or any other drugs.

Stamps will invariably be returned. In all cases so requested, communications will be considered strictly confidential. Reprints of the article, embodying the results of such statistics, will be sent to each correspondent. Address,

DR. H. H. KANE,
*DeQuincy Home, Fort Washington, New York City.*

---

PROFS. D. W. GRAHAM, S. H. Stevenson and M. J. Mergler, of the Woman's Medical College, have been added to the staff of Cook County Hospital. This action of the County Commissioners is to be commended in recognition of the growing usefulness of the Woman's College, as it ranks but second among the medical colleges of Chicago in the number of students sent to the County hospital clinics.

